# *Naja sputatrix* Venom Preconditioning Attenuates Neuroinflammation in a Rat Model of Surgical Brain Injury via PLA2/5-LOX/LTB4 Cascade Activation

**DOI:** 10.1038/s41598-017-05770-7

**Published:** 2017-07-14

**Authors:** Yuechun Wang, Prativa Sherchan, Lei Huang, Onat Akyol, Devin W. McBride, John H. Zhang

**Affiliations:** 1 Department of Physiology & Pharmacology, Loma Linda University School of Medicine, Loma Linda, California, 92354 USA; 2 Department of Physiology, Jinan University School of Medicine, Guangzhou, Guangdong Province China; 3 Department of Anesthesiology, Loma Linda University School of Medicine, Loma Linda, California 92354 USA

**Keywords:** Neurology, Neurological disorders

## Abstract

Inflammatory preconditioning is a mechanism in which exposure to small doses of inflammatory stimuli prepares the body against future massive insult by activating endogenous protective responses. Phospholipase A2/5-lipoxygenase/leukotriene-B4 (PLA2/5-LOX/LTB4) axis is an important inflammatory signaling pathway. *Naja sputatrix* (Malayan spitting cobra) venom contains 15% secretory PLA2 of its dry weight. We investigated if *Naja sputatrix* venom preconditioning (VPC) reduces surgical brain injury (SBI)-induced neuroinflammation via activating PLA2/5-LOX/LTB4 cascade using a partial frontal lobe resection SBI rat model. *Naja sputatrix* venom sublethal dose was injected subcutaneously for 3 consecutive days prior to SBI. We observed that VPC reduced brain edema and improved neurological function 24 h and 72 h after SBI. The expression of pro-inflammatory mediators in peri-resection brain tissue was reduced with VPC. Administration of Manoalide, a PLA2 inhibitor or Zileuton, a 5-LOX inhibitor with VPC reversed the protective effects of VPC against neuroinflammation. The current VPC regime induced local skin inflammatory reaction limited to subcutaneous injection site and elicited no other toxic effects. Our findings suggest that VPC reduces neuroinflammation and improves outcomes after SBI by activating PLA2/5-LOX/LTB4 cascade. VPC may be beneficial to reduce post-operative neuroinflammatory complications after brain surgeries.

## Introduction

Surgical brain injury (SBI) describes the inadvertent injury that occurs to peri-resection brain tissue due to the invasive nature of surgical procedures^1, 2^. During neurosurgical procedures, direct mechanical and heat injuries cause disruption of the blood brain barrier (BBB) which is typified by early vasogenic edema followed by delayed neuronal cell death accompanied by cytotoxic edema^3^. In addition, primary and secondary inflammatory responses further potentiate BBB breakdown resulting in increased intracranial pressure and accompanying higher mortality^3, 4^. Avenues that target neuroinflammation can be an important therapeutic strategy to reduce the consequences of the unavoidable nature of injury that occurs due to surgical manipulation.

Preconditioning has recently emerged as a potential anti-inflammatory strategy and is described as a phenomenon where sublethal inflammatory insults applied to the body triggers anti-inflammatory responses which help develop tolerance against future massive inflammatory events by activating endogenous protective mechanisms^5^. Various preconditioning methods have been shown to be beneficial in ischemic or hemorrhagic stroke models^6–8^. Moreover, prevention of inflammation has been shown to be involved in the mechanism of preconditioning-induced neuroprotection against cerebral and spinal cord ischemia reperfusion injury^9–13^. The elective nature of most neurosurgical procedures provides options to utilize preconditioning strategies as a preventative measure to reduce post-operative complications.

Snakebite envenomation inflicted by most snake species induces local inflammation. Metalloproteinases^14, 15^ and secretory phospholipase A2 (sPLA2)^16, 17^ are important components of snake venoms responsible for venom-induced inflammatory reaction. Secretory PLA2 is the second biggest component of *Naja sputatrix* (Malayan spitting cobra) venom accounting for 15% of its dry weight only second to polypeptide cardiotoxins which comprise 60% of dry weight of the venom^18–20^. Previous study showed that sPLA2 isolated from *Naja sputatrix* venom induced pulmonary inflammation and edema when administered to rats^21^. Mice lacking PLA2 when subjected to cerebral ischemia had smaller infarcts, reduced brain edema and neurological deficits^22^. Additionally, initiation of the arachidonic acid (AA) cascade via PLA2 activation has been shown to occur following transient global ischemia^23^. PLA2 catalyzes the hydrolysis of membrane phospholipids to release AA which is further oxygenated by 5-lipoxygenase (5-LOX) to release leukotriene-B4 (LTB4), a potent attractant for leukocyte recruitment.

In this study, we hypothesized that *Naja sputatrix* venom preconditioning (VPC) will activate the PLA2/5-LOX/LTB4 pathway and provide tolerance against SBI-induced neuroinflammation, thereby reducing brain edema and improving neurological function after SBI.

## Results

There was no mortality during the course of *Naja sputatrix* venom preconditioning using the current regime described in this study. All sham-operated rats survived. The overall mortality in the SBI groups was 16.7%. There was no significant difference in mortality among the experimental SBI groups.

### Venom preconditioning (VPC) reduced brain edema and improved neurological function after SBI

**Figure 1:**
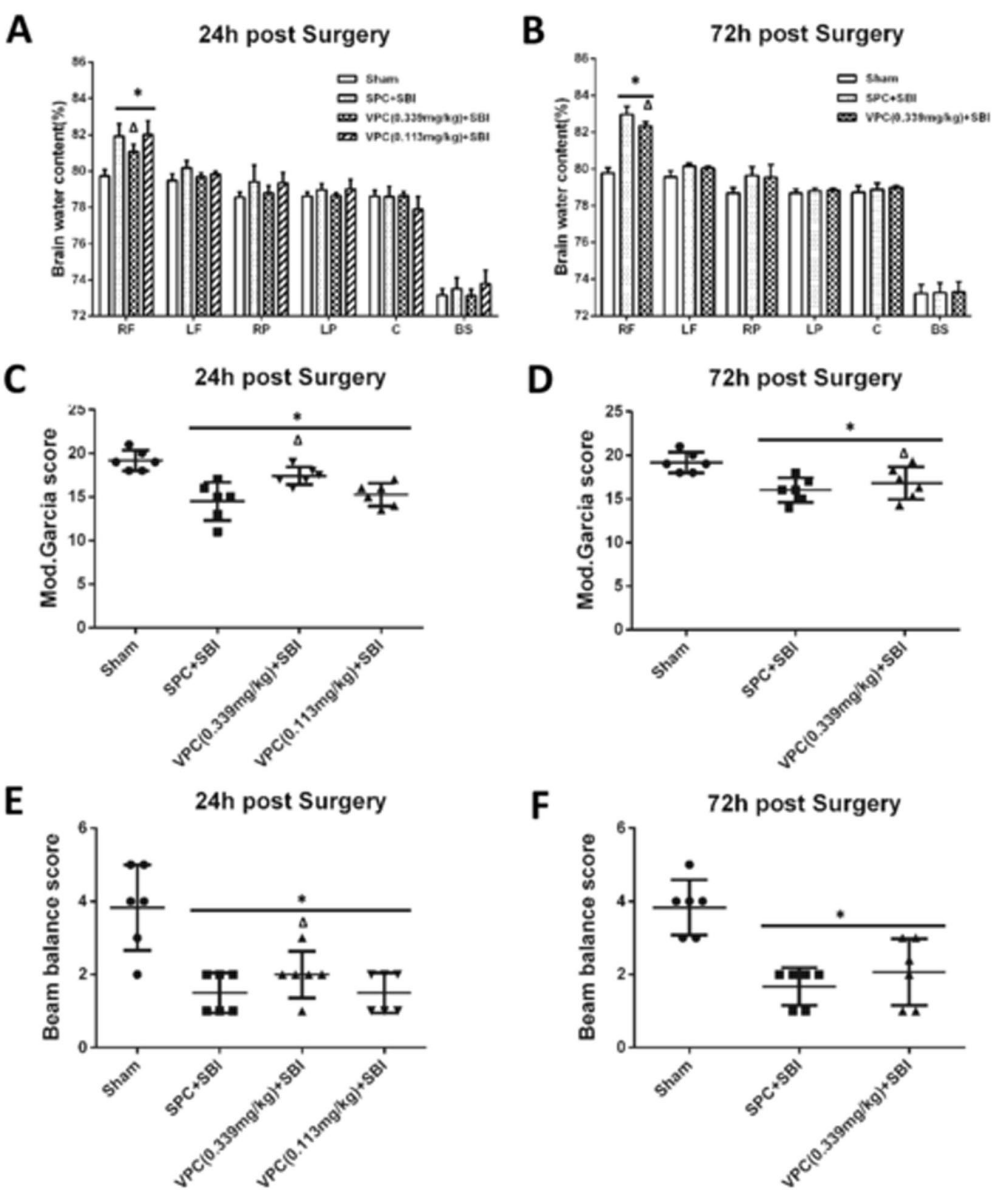
Effects of venom preconditioning (VPC) on brain water content and neurological function 24 h and 72 h after SBI. **(A** and **B)** VPC (0.339 mg/kg) decreased brain water content at the right frontal (RF) peri-resection brain tissue 24 h and 72 h after SBI, respectively. **(C** and **D)** VPC (0.339 mg/kg) improved modified Garcia neurological score 24 h and 72 h after SBI, respectively. **(E)** VPC (0.339 mg/kg) improved beam balance neurological score 24 h after SBI. **(F)** VPC (0.339 mg/kg) showed a trend to improved beam balance score 72 h after SBI. *p < 0.05 vs Sham, ^∆^p < 0.05 vs SPC + SBI. Data are shown as mean ± SD. n = 6–8/group. RF = right frontal lobe, LF = left frontal lobe, RP = right parietal lobe, LP = left parietal lobe, C = cerebellum, BS = brain stem.

The brain water content (BWC) was increased and neurological function was worsened at 24 and 72 h after SBI compared to the sham-operated rats. High dose VPC (0.339 mg/kg) significantly reduced BWC in the right frontal peri-resection brain tissues at 24 and 72 h after SBI compared to saline preconditioning (SPC) group (Fig. 1A and B, respectively) and improved modified Garcia neurological scores at both time points after SBI (Fig. 1C and D, respectively). High dose VPC (0.339 mg/kg) improved beam balance scores compared to SPC group at 24 h after SBI but not at 72 h (Fig. 1E and F, respectively). Low dose VPC (0.113 mg/kg) did not produce any significant difference in BWC or neurological function compared to SPC group.

### Venom preconditioning (VPC) induced edema and local skin inflammation at the subcutaneous injection site

**Figure 2:**
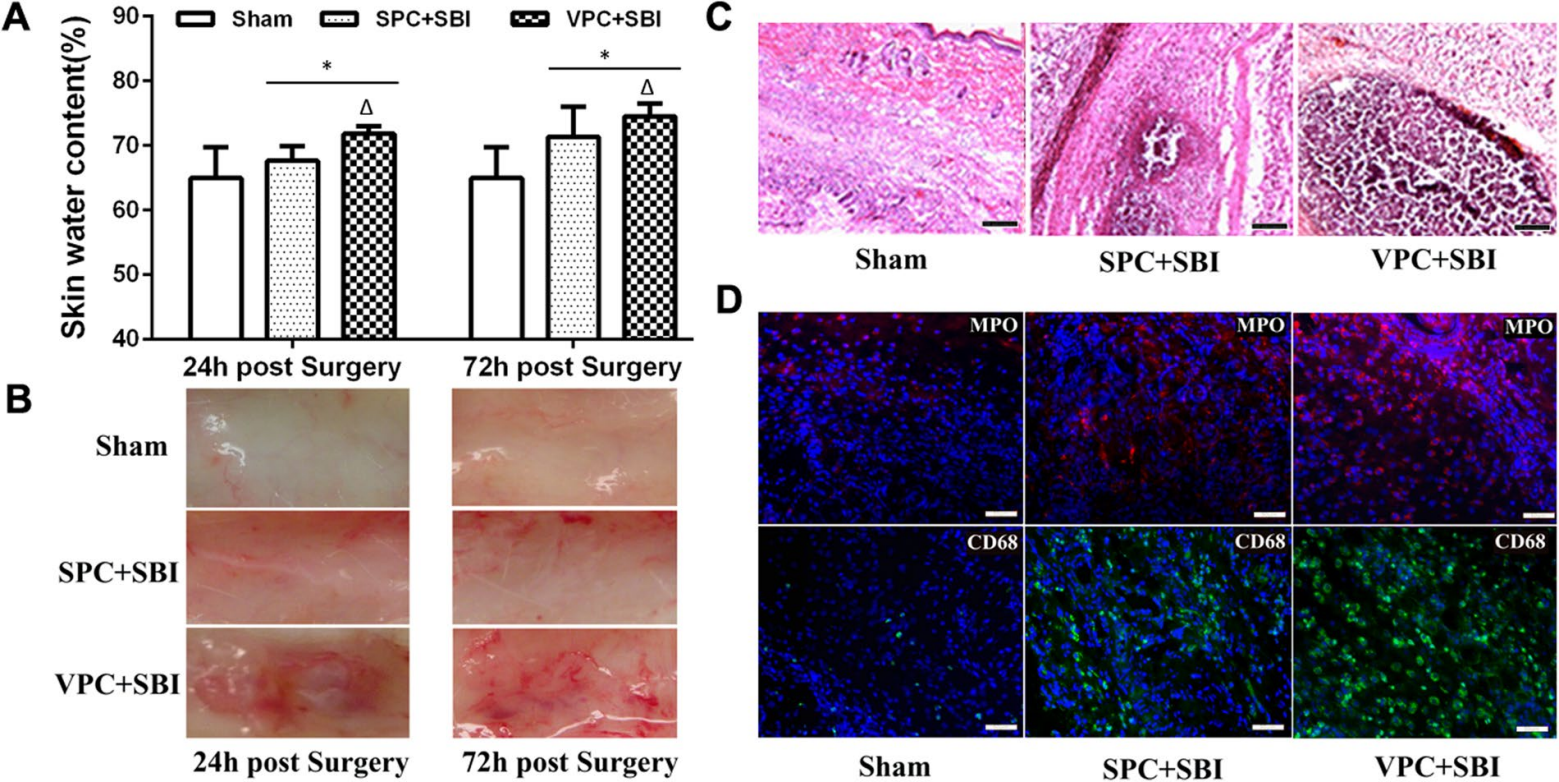
Venom preconditioning (VPC) induced local skin changes at the subcutaneous injection site. **(A)** Skin water content was increased in VPC + SBI group compared to Sham and SPC + SBI groups 24 h and 72 h after SBI. *p < 0.05 vs Sham, ^∆^p < 0.05 vs SPC + SBI. Data are shown as mean ± SD. n = 8/group. **(B)** VPC induced local inflammation at the skin injection site. Subcutaneous region showed apparent redness, edema and congestion in VPC + SBI group compared to Sham and SPC + SBI groups. n = 3/group. **(C)** Hematoxylin and eosin (H&E) staining of skin samples showing neutrophil infiltration in VPC + SBI group compared to Sham and SPC + SBI groups 24 h after SBI. n = 3/group. Scale bar = 100 μm. **(D)** Immunofluorescence staining of skin samples showing expression of neutrophil marker myeloperoxidase (MPO) (Texas Red-red) and macrophage/microglia marker (CD68) (FITC-green) with DAPI (blue) in VPC + SBI group compared to Sham and SPC + SBI groups 24 h after SBI. n = 3/group. Scale bar = 50 μm.

The skin water content was significantly increased in the VPC rats compared to SPC rats and sham rats (without any skin injection) at 24 h and 72 h (Fig. 2A). The skin site used for subcutaneous injections for VPC showed apparent redness, edema and congestion (Fig. 2B). Hematoxylin and eosin (H&E) staining of skin samples showed increased neutrophil infiltration in VPC + SBI group compared to Sham and SPC + SBI groups 24 h after SBI, though it was not quantified (n = 3/group) (Fig. 2C). Immunofluorescence staining of skin samples showed increased expression of neutrophil marker myeloperoxidase (MPO) and macrophage/microglia marker (CD68) in VPC + SBI group compared to Sham and SPC + SBI groups, though it was not quantified (n = 3/group) (Fig. 2D).

### Venom preconditioning (VPC) increased peripheral white blood cell (WBC) count 24 h after SBI

**Figure 3:**
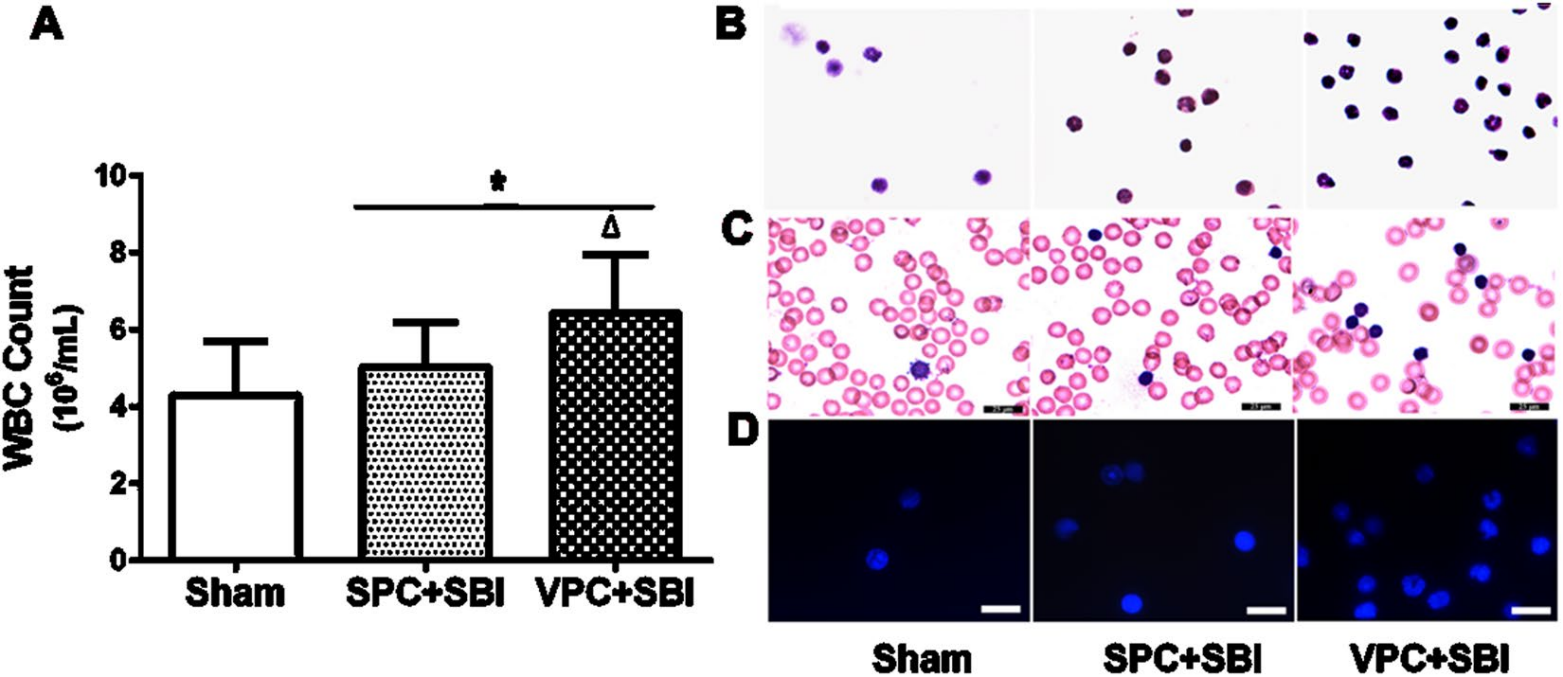
Total peripheral WBC count and peripheral WBC suspension staining 24 h after SBI. **(A)** Total peripheral WBC count was increased in VPC + SBI group compared to Sham and SPC + SBI groups. *p < 0.05 vs Sham, ^∆^p < 0.05 vs SPC + SBI. Data are shown as mean ± SD. n = 8/group. **(B)** Hematoxylin and eosin (H&E) staining (n = 3/group) **(C)** Wrights staining (n = 3/group) and **(D)** DAPI staining (n = 3/group) of peripheral WBC suspension showing segmented granulocytes in VPC + SBI group. All scale bars = 25 μm.

Peripheral WBC count was significantly higher in VPC + SBI rats compared to sham and SPC + SBI group (Fig. 3A). Peripheral WBC smear showed an increase in segmented granulocytes in VPC + SBI rats compared to sham and SPC + SBI rats 24 h after SBI using H&E, Wrights and DAPI staining (Fig. 3B–D, respectively), though the cell numbers were not quantified (n = 3/group).

### Inflammatory markers were increased at the peri-resection site 24 h after SBI

Double immunofluorescence staining of brain sections from the SPC + SBI rats showed that 5-LOX was predominantly expressed by NeuN-positive neurons and to a lesser extent by the GFAP-positive astrocytes but not by the IBa-1 labeled microglia (Supplementary Fig. S1) at the peri-resection site 24 h after SBI. Likewise, brain sections from SPC + SBI rats showed cells positively stained with inflammatory markers CD68, MPO and IL1 were distributed in the peri-resection tissue surrounding the resection site (Supplementary Fig. S2A) and at further distance from the resection site (Supplementary Fig. S2B) 24 h after SBI. Western blot of brain samples from SPC + SBI rats showed that the expression of GFAP, CD68 and 5-LOX was increased at the right frontal peri-resection site compared to the contralateral left frontal counterpart (Supplementary Fig. S2C) 24 h after SBI.

### Venom preconditioning (VPC) decreased neuroinflammation at the peri-resection site 24 h after SBI

**Figure 4:**
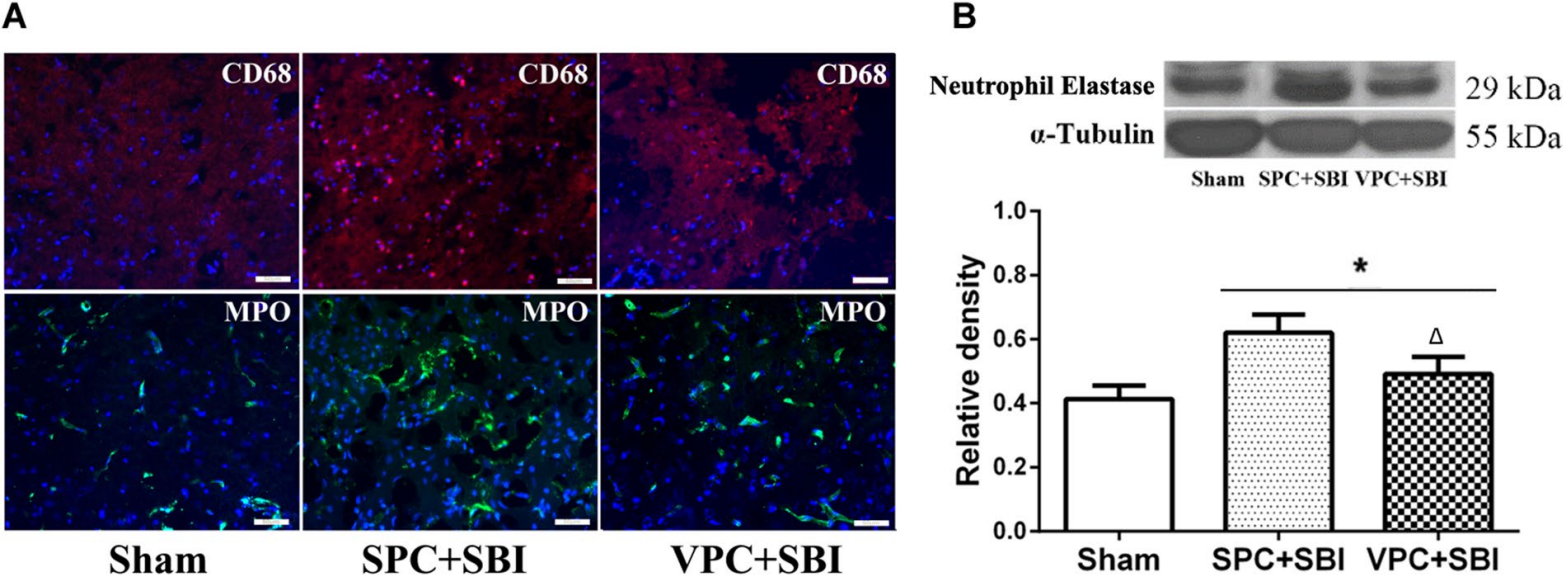
Immunofluorescence staining and western blot showing inflammatory markers in the right frontal peri-resection site in Sham, SPC + SBI and VPC + SBI groups 24 h after SBI. **(A)** Immunofluorescence staining showed fewer CD68- and MPO-positive cells in VPC + SBI rats compared to SPC + SBI group. n = 2/group. Scale bar = 50 μm. **(B)** Western blot quantification showed that neutrophil elastase was reduced in VPC + SBI group compared to SPC + SBI group. *p < 0.05 vs Sham, ^∆^p < 0.05 vs SPC + SBI. Data are shown as mean ± SD. n = 6/group. The full length western blot pictures are shown in Supplemental Figure S3.

Immunofluorescence staining showed fewer CD68 and MPO-positive cells at the peri-resection site in VPC + SBI rats compared with SPC + SBI rats (Fig. 4A), though it was not quantified (n = 2/group). Western blot quantification showed that the expression of neutrophil elastase was reduced in VPC + SBI rats compared to SPC + SBI group (Fig. 4B).

### Manoalide and Zileuton reversed venom preconditioning (VPC)-induced anti-inflammatory effects 24 h after SBI

**Figure 5:**
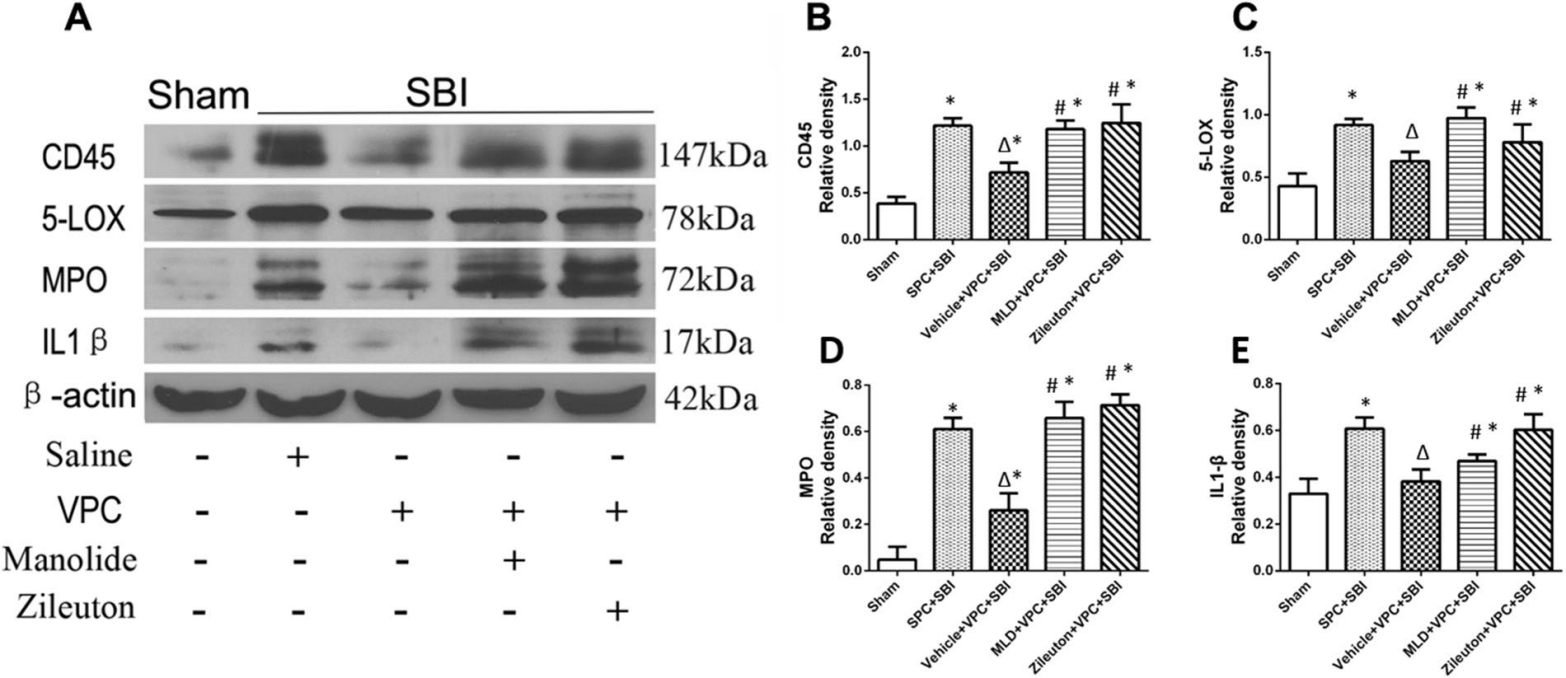
Venom preconditioning reduced inflammatory markers at the right frontal peri-resection site 24 h after SBI, which was reversed with Manoalide and Zileuton. **(A)** Representative western blot bands showing the changes in expression of inflammation-associated markers CD45, 5-LOX, MPO and IL-1 at the peri-resection site 24 h after SBI. The expression of CD45 **(B)**, 5-LOX **(C)**, MPO **(D)** and IL-1β **(E)** were reduced in VPC + SBI rats compared to SPC + SBI group. Manoalide and Zileuton reversed the effects of VPC on the expression of all the inflammatory markers. *p < 0.05 vs Sham, ^∆^p < 0.05 vs SPC + SBI, ^#^p < 0.05 vs VPC + SBI. Data are shown as mean ± SD. n = 6/group. The full length western blot pictures are shown in Supplemental Figure S4.

Western blot showed that the expression of pro-inflammatory markers CD45 (Fig. 5B), 5-LOX (Fig. 5C), MPO (Fig. 5D), and IL-1β (Fig. 5E) were significantly increased in the peri-resection brain tissue in the SPC + SBI group compared to sham. VPC significantly reduced the expression of CD45, 5-LOX, MPO, and IL-1β in the peri-resection brain tissue compared to SPC group, whereas Manoalide and Zileuton reversed the protective effects of VPC (Fig. 5A–E, respectively).

### Manoalide and Zileuton reversed the effects of venom preconditioning (VPC) on peripheral WBC count and blood LTB4 levels 24 h after SBI

**Figure 6:**
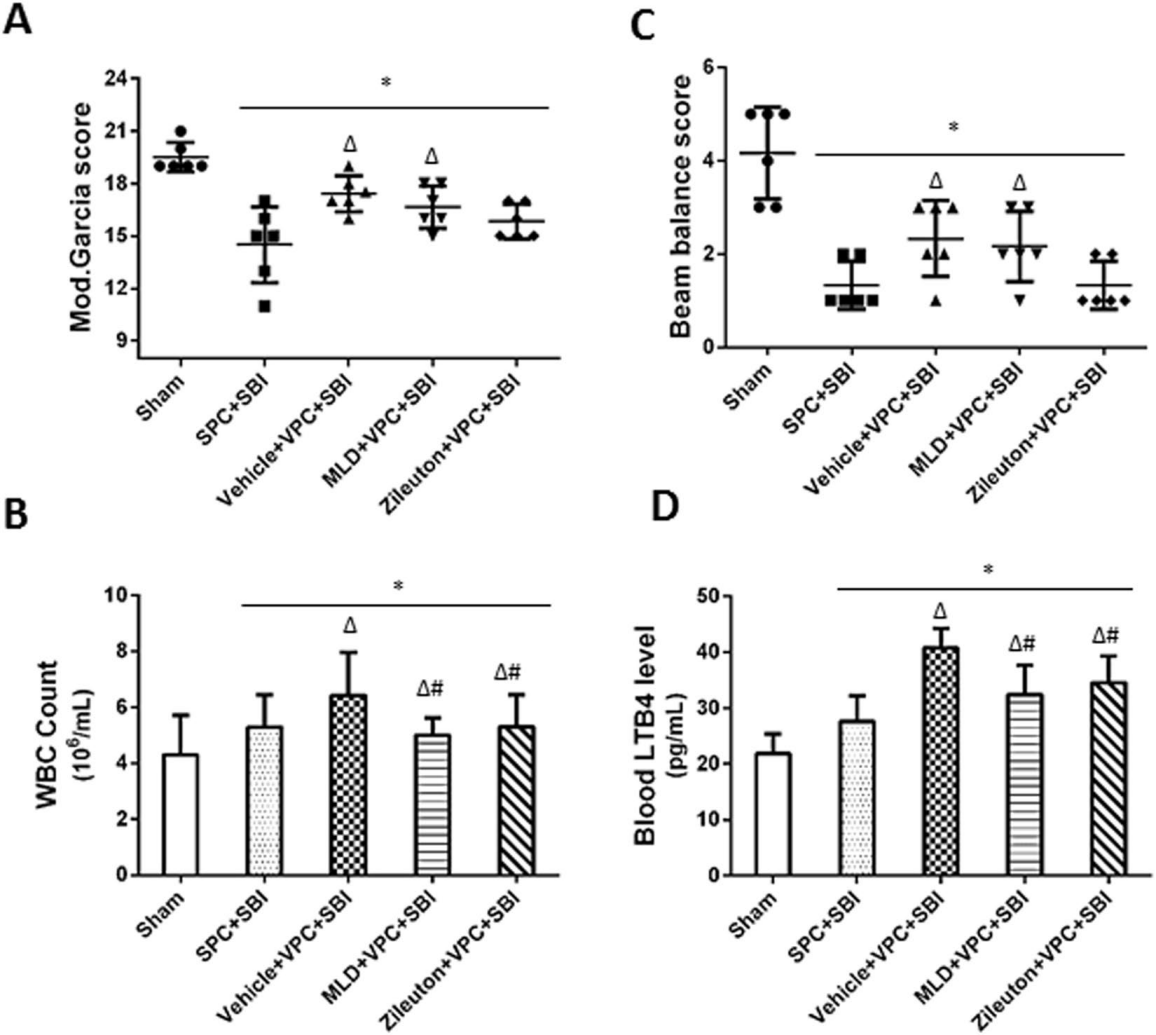
Effects of Manoalide and Zileuton administration with venom preconditioning (VPC) on neurological scores, peripheral WBC count and blood LTB4 level 24 h after SBI. VPC improved modified Garcia neurological score **(A)** and beam balance score **(C)** compared to SPC + SBI group. Manoalide and Zileuton did not reverse the beneficial effect of VPC on neurological scores. The peripheral WBC count **(B)** and blood LTB4 level **(D)** was increased in VPC + SBI group compared to SPC + SBI, which was reversed with Manoalide and Zileuton. *p < 0.05 vs Sham, ^∆^p < 0.05 vs SPC + SBI, ^#^p < 0.05 vs VPC + SBI. Data are shown as mean ± SD. n = 6/group.

Neurological function was significantly improved in VPC + SBI group compared to SPC + SBI group evaluated using modified Garcia neurological test (Fig. 6A) and beam balance test (Fig. 6C). Both Manoalide and Zileuton did not counter the beneficial effects of VPC on SBI-induced neurological deficits. The peripheral WBC count (Fig. 6B) and blood LTB4 levels (Fig. 6D) were increased in VPC + SBI rats compared to SPC + SBI group which were both reversed with Manoalide and Zileuton (Fig. 6B and D, respectively).

### Venom preconditioning (VPC) showed no significant cardiotoxic effects

**Figure 7:**
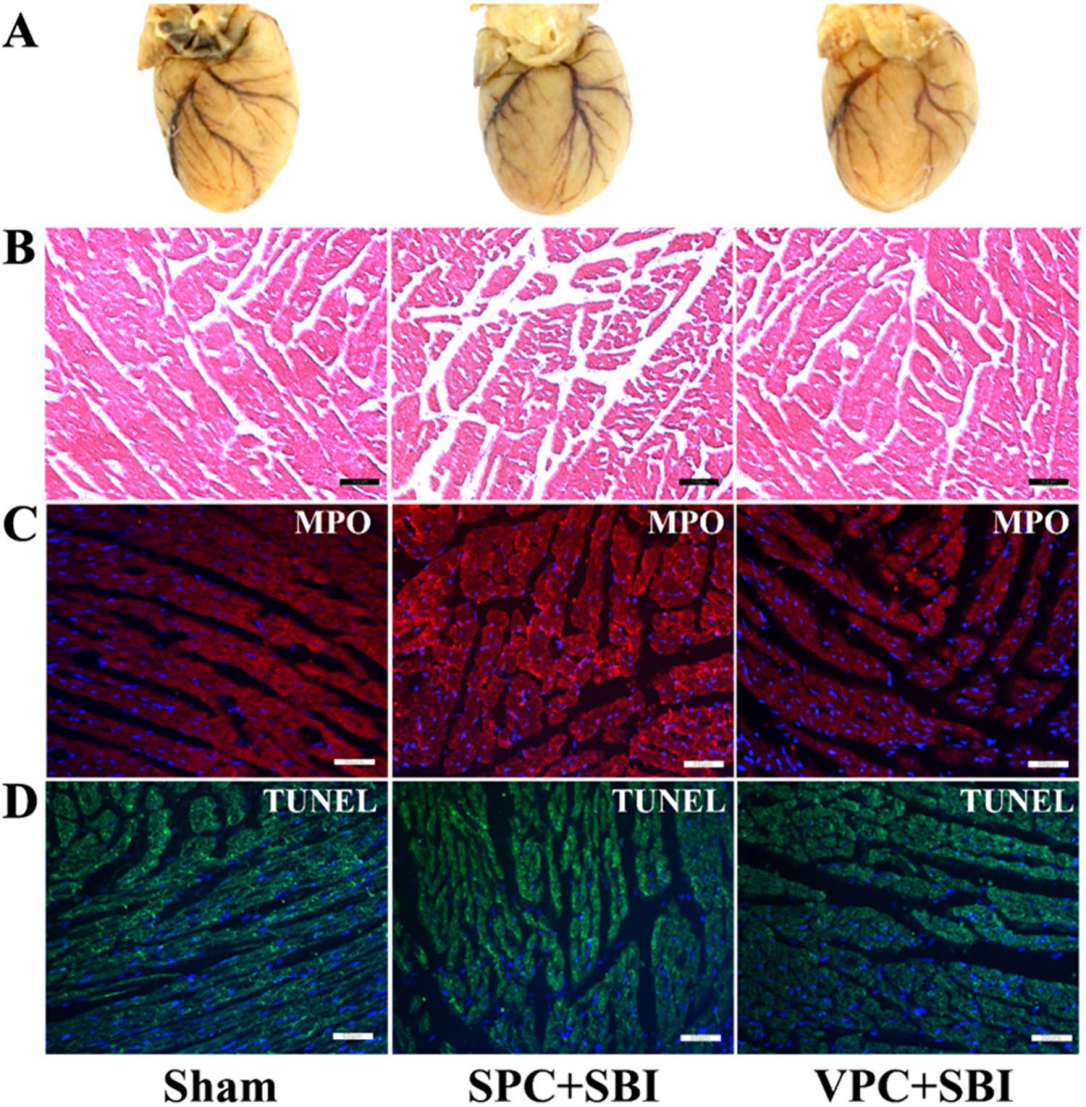
Effect of venom preconditioning (VPC) on the cardiac muscles 24 h after SBI. **(A)** Gross cardiac shape, **(B)** H&E staining, scale bar = 10 μm, **(C)** MPO staining, scale bar = 50 μm and **(D)** TUNEL staining, scale bar = 50 μm. There was no significant difference in cardiac morphology in the SPC + SBI and VPC + SBI groups compared to Sham. All the pictures are representative of 3 animals per group.

There was no significant difference in cardiac morphology among VPC + SBI or SPC + SBI groups compared to sham which was evaluated by gross cardiac examination (Fig. 7A), H&E staining (Fig. 7B), MPO staining (Fig. 7C) and TUNEL staining (Fig. 7D) at 24 h after SBI.

## Discussion

In this study, we evaluated whether *Naja sputatrix* venom preconditioning would be beneficial for neurosurgical injury using an SBI rat model. We made the following observations in our study: (1) *Naja sputatrix* venom preconditioning (VPC) for 3 consecutive days at a dose of 0.339 mg/kg per day attenuated SBI-induced brain edema and improved neurological function 24 and 72 h after SBI. (2) VPC elicited peripheral inflammatory response characterized by an increase in peripheral WBC count as well as characteristic inflammatory response at the subcutaneous skin injection site. (3) VPC reduced neutrophil infiltration and inflammatory cytokine release in the peri-resection brain tissue 24 h after SBI. (4) The expression of 5-LOX in the peri-resection brain tissue was elevated 24 h after SBI which was reduced with VPC. (5) The selective PLA2 inhibitor, Manoalide and 5-LOX inhibitor, Zileuton reversed the protective effects of VPC against SBI-induced neuroinflammation 24 h after SBI. (6) The VPC regime used in this study including the dose, timing and subcutaneous administration of the venom did not elicit any significant adverse cardiotoxic effects.

We observed that 3 days of venom preconditioning reduced brain edema, neuroinflammation and improved post-operative neurological function after SBI. Venom preconditioning induced a mild peripheral inflammatory response at the subcutaneous injection site on the skin which presented with redness and edema. Previous studies show that cobra venom toxins have high affinity for tissues at the site of injection^24^. The injection associated mechanical injury and hemolytic components from the snake venom causes minor bleeding leading to Ca^2+^ release and leukocyte extravasation from blood vessels into the skin injection site. In the presence of Ca^2+^, venom PLA2 hydrolyzes membrane glycerophospholipids at the sn-2 ester to release arachidonic acid (AA) which is further converted into the chemoattractant leukotriene B4 (LTB4) in the presence of the enzyme leukocyte-derived 5-lipoxygenase (5-LOX)^25, 26^. Small molecules AA and LTB4 have high lipid solubility and low molecular mass that favors passage across the BBB^27, 28^. Consequently, a concentration gradient is established with higher levels of small molecular AA and LTB4 in the peripheral circulation and low concentration in the brain. Accordingly, we observed that VPC rats had an elevated blood LTB4 levels.

Aggregation and activation of leukocytes has been associated with increased severity of inflammation and secondary damage following CNS injury^29–31^. Likewise, peripheral immune cell infiltration at the perisurgical site has been shown to aggravate neuroinflammation after SBI^1, 2^. We hypothesized that peripheral inflammatory response induced by preconditioning with sublethal doses of venom injections would elicit endogenous protective mechanisms to prepare against SBI-induced neuroinflammation. Previous studies show that PLA2/5-LOX/LTB4 cascade is activated after neurological injuries. Following traumatic or ischemic brain injuries, direct mechanical insult and successive calcium influx activate PLA2^32, 33^ and expression of 5-LOX in the brain was markedly increased^34–36^. Likewise, LTB4 was elevated at 4 hours and peaked at 24 hours after traumatic brain injury in rats^37^. LTB4 was shown to be involved in the pathogenesis of spinal cord injury through amplification of leukocyte infiltration^38^. Additionally, leukocytes infiltrated after brain injury can increase LTB4 production from neurons and astrocytes via transcellular mechanism^39, 40^ which can further augment leukocyte transmigration leading to a vicious cycle. We hypothesized that the PLA2/5-LOX/LTB4 cascade is amplified with *Naja sputatrix* venom preconditioning, since 15% of the dry weight of the venom is composed of PLA2^18–20^. In the presence of increased peripheral LTB4 with venom preconditioning, leukocytes get trapped in the peripheral circulation and at the injection site. The primary role of leukocytes recruited to the inflamed venue is to destroy invading pathogens and dead cells by granular enzymes and oxygen species^41, 42^. However, peripherally accumulated leukocytes undergo spontaneous or death receptor-induced apoptosis shortly after physiological maturation or pathological activation^43–47^. In accordance, we observed that leukocyte infiltration was reduced at the peri-resection brain tissue in SBI rats following 3 days of venom preconditioning, which was possibly due to peripheral depletion of leukocytes with venom preconditioning.

Based on our findings, we suggest that venom preconditioning induced peripheral PLA2/5-LOX/LTB4 activation that may have executed an inhibitory feedback on the central PLA2/5-LOX/LTB4 cascade following SBI, thereby reducing leukocyte infiltration into the injured brain. Our results showed that expression of 5-LOX in the brain was decreased after 3 days of venom preconditioning and was accompanied by reduced leukocyte infiltration and inflammatory marker expression at the peri-resection site. This translated into reduced brain edema and improved neurological function in venom preconditioned SBI rats. Furthermore, we administered the selective pharmacological inhibitors, Manoalide and Zileuton with venom preconditioning to block the activities of PLA2 and 5-LOX. Our results showed that both interventions reversed the effects of venom preconditioning which was reflected by reduced peripheral WBC count and blood LTB4 levels and increased expression of inflammatory markers after SBI. These results suggest that venom preconditioning-induced neuroprotection was in part mediated through the activation of peripheral PLA2/5-LOX/LTB4 cascade.

Our study had some limitations. First, snake venom is composed of numerous components including PLA2, which may have played a role in the effects that we observed in our study. Additionally, pleotropic anti-inflammatory effects in venom preconditioning could be involved in preconditioning induced protection against SBI. We did not isolate the venom fractions to identify the protein responsible for the preconditioning effects. Further studies are required to test the purified venom components to elucidate the component that mediates the anti-inflammatory effects during venom preconditioning for clinical translation. Second, the lack of a PLA2 preconditioning group is a limitation in this study. We hypothesized that PLA2 was the active component in the snake venom to produce anti-inflammatory effects during venom preconditioning. However, in this study we did not test the effects of PLA2 preconditioning. Studies using PLA2 preconditioning is currently being pursued. Third, the effects of venom preconditioning could be due to a combination of both local and systemic inflammatory effects of the venom and venom components. It is possible that venom PLA2 may have entered the circulation and directly exerted peripheral effects. In addition to mild inflammatory changes at the subcutaneous injection site, the peripheral WBC count was increased with venom preconditioning which could possibly be an effect of PLA2 entry into the circulation. Fourth, we did not measure the effect of Manoalide on venom or endogenous PLA2 activity. The method we used to inhibit venom PLA2 activity was based on previous publications^48–51^ which showed that the protocol we used was an effective *ex vivo* chemical method to inactivate PLA2. Moreover, the dose of Manoalide we used was very low compared to the *in vivo* dose that was required to inhibit endogenous PLA2 in rats^52^, and therefore may not have reached a pharmacological blood concentration to inhibit endogenous PLA2 activity. However, further studies are needed using sPLA2/cPLA2/iPLA2-specific inhibitors to test whether the endogenous or venom PLA2s are affected. Fifth, we used a small sample size consisting of only 2–3 animals per group for immunostaining experiments and therefore, did not quantify positively stained cells. A lack of quantification of the immunostaining experiments is a limitation of this study. Lastly, we did not evaluate effects of the venom on other peripheral organs. The systemic effects of venom require detailed exploration in future studies. Polypeptide cardiotoxins constitute 60% of dry weight of the *Naja sputatrix* venom^20^ and has been reported to induce cardiotoxic effects manifesting as gene profiles changes involved in inflammation, apoptosis, ion transport and energy metabolism^53^. No adverse cardiac effects were observed with the preconditioning regime used in this study. Adverse effects of venom should be carefully considered when designing preconditioning regimes.

Of note, venom preconditioning may have context-dependent effects where the timing, duration, dosage, administration routes, and types of snake venom exposure can result in diverse effects. Therefore, it is imperative to measure blood biomarkers such as WBC count, LTB4 levels and PLA2 activity to optimize the desirable preconditioning effects of the venom specific to each individual. We tested the preconditioning effects of two doses of *Naja sputatrix* venom (0.339 mg/kg and 0.113 mg/kg). Since our initial outcome studies showed that 0.339 mg/kg was effective in reducing brain edema and improved neurological function in SBI rats, we continued to test this dose for further studies and did not test higher doses of the venom. Even though there was only a 3-fold difference in the two doses of the venom that we tested, given the potent toxic effects of *Naja sputatrix* venom, it is possible that a 3-fold difference in concentration is probably enough to induce significant differences when injected into animals. Additionally, previous study showed that the cytolytic activity of *Naja sputatrix* venom *in vitro* increased when the venom concentration was increased by 2 fold^52^. Higher dose of venom preconditioning could be more effective but may also lead toxic side effects or mortality.

In summary, our findings demonstrate that *Naja sputatrix* venom preconditioning attenuated neuroinflammation after SBI by impairing peripheral leukocyte trafficking to the injury site by reducing 5-LOX activity possibly through a negative feedback provided by activation of peripheral PLA2/5-LOX/LTB4 cascade. In addition, the preconditioning dose of venom did not elicit any toxic effects. Additional studies are needed to better understand toxicities and side effects of venom preconditioning for clinical translation.

## Methods

### Animals

All procedures were approved by Institutional Animal Care and Use Committee at Loma Linda University following NIH Guide for Care and Use of Laboratory Animals. Adult male Sprague-Dawley rats, 260 to 300 g, were used in the study.

### Experimental Design

#### Experiment 1

Thirty-two rats were randomly assigned into four groups (n = 8/group): Sham, SPC + SBI, VPC (0.339 mg/kg) + SBI, and VPC (0.113 mg/kg) + SBI. *Naja sputatrix* venom (Sigma Aldrich, St. Louis, MO) was dissolved in normal saline to obtain 20 mg/mL stock solution which was further diluted to make a 0.5 mg/mL working solution. The dose of venom to inject per rat was determined based on the body weight, and the corresponding volume was taken from the working solution to inject to the rats. The fur on the nape of the neck was shaved and a rectangular area 2 × 2 cm was outlined with a marker. Venom or saline was injected subcutaneously at the same depth for 3 consecutive days in different spots that were included within the 2 × 2 cm rectangular area in the nape of the neck. The dose and route were chosen based on previous publications^18, 19^. SBI was induced by partial resection of right frontal lobe 24 h after last preconditioning injection. Neurological function was evaluated 24 h after SBI and brain samples were collected for BWC analysis. The effective dose established in this experiment was used further.

Next, twenty-four rats were randomly assigned into three groups (n = 8/group): Sham, SPC + SBI, and VPC (0.339 mg/kg) + SBI. Neurological function was evaluated 72 h after SBI and brain samples were collected for BWC analysis.

#### Experiment 2

Twenty-four rats were divided into three groups (n = 8/group): Sham, SPC + SBI, and VPC (0.339 mg/kg) + SBI. Rats were sacrificed at 24 h to collect skin samples, blood samples, and brain samples for western blot and immunohistochemistry. Skin samples were obtained by resecting the skin along margins of the rectangle.

#### Experiment 3

Thirty rats were divided into five groups (n = 6/group): Sham, SPC + SBI, Vehicle + VPC + SBI, Manoalide + VPC + SBI, and Zileuton + VPC + SBI. The PLA2 inhibitor, Manoalide (Cayman Chemical, Ann Arbor, MI) was dissolved in ethanol to obtain a stock solution with concentration of 4.8 mM. Manoalide was added to *Naja sputatrix* venom 1:20 and incubated at 42 °C for 40 mins to inactivate PLA2^48–51^. The PLA2 inactivated venom was injected subcutaneously in the nape of the neck for 3 consecutive days. The selective 5-LOX inhibitor, Zileuton (Santa Cruz Biotechnology, Santa Cruz, CA) was dissolved in ethanol to obtain 20 mg/mL stock solution. Zileuton (0.5 mg/kg) was injected subcutaneously, in a different spot that was included within the same 2 × 2 cm rectangular area in the nape of the neck, 30 mins before each VPC injections^54–57^. Brain samples were collected 24 h after SBI for western blot analysis.

### Surgical Brain Injury Rat Model

Rats were subjected to SBI as previously described^2, 56^. Under isoflurane anesthesia, the skin was incised to expose the skull. A bone window 5 × 5 mm was drilled in right frontal skull 2 mm lateral to sagittal suture and 1 mm proximal to coronal suture. The bone flap was removed and dura was incised to visualize the right frontal lobe. The frontal lobe was incised along margins of bone window to perform a partial frontal lobe resection. The depth of resection was extended till base of skull was visible. Hemostasis was ensured and skin was sutured. Sham rats underwent the same surgical procedure to remove the bone flap but dura and frontal lobe was kept intact. Normal saline 1 ml was injected subcutaneously for fluid replacement. All animals were injected buprenorphine (0.03 mg/kg) once subcutaneously in the right flank at the end of surgery for post-operative analgesia.

### Measurement of Brain Water Content (BWC)

The animals were decapitated under deep anesthesia and brains were quickly extracted and dissected into 6 parts: right frontal, left frontal, right parietal, left parietal, cerebellum and brain stem. Wet weights were measured immediately and weighed again after drying brain samples in 105 °C oven for 48 h. BWC of each brain region was calculated using the formula: [(wet weight − dry weight)/wet weight] × 100% as previously described^56^.

### Assessment of Neurological Function

Modified Garcia test evaluated seven parameters including spontaneous activity, side stroking, vibrissae touch, limb symmetry, climbing, lateral turning and forelimb walking as described previously^2, 56^. The maximum score was 21. The beam balance test^2, 58^ examined motor coordination, balance and proprioception. Rats were placed on the middle of a 90 cm × 2.25 cm beam and observed for 1 min. Distance traveled on the beam, duration stayed on the beam, time to reach platform at the end of the beam were recorded based on which scores were given ranging from 0 to 5. Higher scores indicated better neurological function.

### Measurement of White Blood Cell (WBC) Count

Blood sample 200 μL was drawn from left ventricle during sacrifice and mixed with 800 μL of citrate-phosophate-dextrose solution. Red blood cells (RBC) was removed from blood samples by adding 10 mL RBC lysis buffer at room temperature for 30 mins followed by centrifugation at 1500 rpm for 5 mins and removal of the supernatant^59^. This step was repeated 3 times to obtain WBC pellet. The WBC pellet was resuspended with 1 mL of 0.01 M phosphate buffered saline (PBS) to obtain WBC suspension. WBC count was determined using TC10 TM Automated Cell Counter (Bio-Rad, Hercules, CA)^60^.

### Wrights Staining

A thin film of WBC suspension was smeared on microscope slide. The air dried slides were dipped in One Step Wrights Stain (Polysciences, Inc., Warrington, PA) for 15–30 seconds, then in deionized water for 25 seconds twice followed by quick dips in deionized water. Slides were dried after which immersion oil was applied and observed under microscope (Olympus BX51).

### Hematoxylin and Eosin (H&E) Staining

The slides were immersed in 95% Flex and 70% Flex for 1 min, respectively; rinsed in tap water and then distilled water; stained for nuclei with hematoxylin for 1–2 mins; rinsed in tap water; differentiated with 0.3% acid alcohol; rinsed in tap water; rinsed in Scott’s tap water substitute; rinsed in tap water; stained with eosin for 20 seconds to 1 min; dehydrated, cleared and mounted. Microphotographs were taken using a microscope (Olympus BX51).

### Western Blot Assay

Equal amounts of protein (30 μg) from right frontal peri-resection site were separated by 10% SDS-PAGE and transferred onto nitrocellulose membrane (Bio-Rad, Hercules, CA) as previously described^61, 62^. The primary antibodies included anti-GFAP (1:2,000) and anti-CD68 (1:1,000) (both from Santa Cruz Biotechnology, Santa Cruz, CA), anti-5LOX (1:1,000, Abcam, Cambridge, MA), anti-neutrophil elastase (1:1,000), anti-CD45 (1:1,000) and anti-myeloperoxidase (MPO) (1:1,000) (all from Santa Cruz Biotechnology, Santa Cruz, CA), anti-IL-1β (1:1.000, Abcam, Cambridge, MA). The same membranes were probed with anti-β-actin (1:5,000, Santa Cruz Biotechnology, Santa Cruz, CA) as loading controls. Appropriate secondary antibodies (1:4,000, Santa Cruz Biotechnology, Santa Cruz, CA) were incubated for 1 h at room temperature, membranes were exposed using a chemiluminescence reagent kit (ECL Plus, Amersham Biosciences, Arlington Heights, IL) and X-ray films were developed. The band density was quantified using Image J software (NIH, Bethesada, MA).

### Immunofluorescence Staining

Sections were incubated overnight at 4 °C as previously described^1^ with the following primary antibodies: anti-5LOX (1:150, Santa Cruz Biotechnology, Santa Cruz, CA) co-stained with anti-NeuN (1:200, Millipore, Billerica MA), anti-GFAP (1:400, Santa Cruz Biotechnology, Santa Cruz, CA) and anti-IBa1 (1:200, Abcam, Cambridge, MA). Sections were also incubated with anti-MPO (1:100), anti-CD68 (1:100), anti-IL1β (1:150) antibodies (all from Santa Cruz Biotechnology, Santa Cruz, CA) followed by incubation with FITC- or Texas red-conjugated secondary antibodies (Jackson Immuno Research, West Grove, PA). Slides were covered DAPI and visualized under a fluorescence microscope (Olympus BX51).

### ELISA for Blood LTB4 Levels

Blood LTB4 levels were measured using rat leukotriene B4 (LTB4) Elisa kit (MyBioSource, San Diego, CA). Standards or samples 50 μL were added to antibody pre-coated microtiter plate. Next, conjugate 100 μL, which consisted of the horseradish peroxidase (HRP)-conjugated polyclonal antibody provided with the kit, was added to each well and incubated for 1 h at 37 °C. The microtiter plate was washed four times. Next, 50 μL substrate A was added followed by 50 μL substrate B and incubated for 15 mins at 20–25 °C in the dark. Lastly, 50 μL stop solution was added and optical density was measured at 450 nm using a microtiter plate reader. According to the vendor specification provided with the ELISA kit, the sensitivity limit for the assay kit was 1.0 pg/mL. We first made a standard curve ranging from 0 pg/mL to 1000 pg/mL. The blood LTB4 levels were determined using the standard curve.

### Terminal Deoxynucleotidyl Transferase Biotin-dUTP Nick End Labeling (TUNEL) Staining

TUNEL assay kit (Roche, Mannheim, Germany) was used as previously described^63^. Briefly, slides were soaked in 0.5% Triton-100 at room temperature for 15 mins, washed twice in PBS, and then incubated with TUNEL reaction mixture at 37 °C for 90 mins. Slides were covered with DAPI and then observed under a fluorescence microscope (Olympus BX51).

### Statistical Analysis

Statistical analysis was performed using Sigma Plot 10. Quantitative data were expressed as mean ± SD. The statistical differences among groups were determined by One-way ANOVA for multiple comparisons and Student-Newman-Keuls post hoc test. Statistical significance was set at p < 0.05.

## Electronic supplementary material

Supplementary Information
