# Author Correction: *Naja sputatrix* Venom Preconditioning Attenuates Neuroinflammation in a Rat Model of Surgical Brain Injury via PLA2/5-LOX/LTB4 Cascade Activation

**DOI:** 10.1038/s41598-025-88937-x

**Published:** 2025-02-26

**Authors:** Yuechun Wang, Prativa Sherchan, Lei Huang, Onat Akyol, Devin W. McBride, John H. Zhang

**Affiliations:** 1 Department of Physiology & Pharmacology, Loma Linda University School of Medicine, Loma Linda, California 92354 USA; 2 Department of Physiology, Jinan University School of Medicine, Guangzhou, Guangdong Province China; 3 Department of Anesthesiology, Loma Linda University School of Medicine, Loma Linda, California 92354 USA

Correction to: *Scientific Reports* 10.1038/s41598-017-05770-7, published online 14 July 2017

This Article contains an error in Figure 7.

**Fig. 7:**
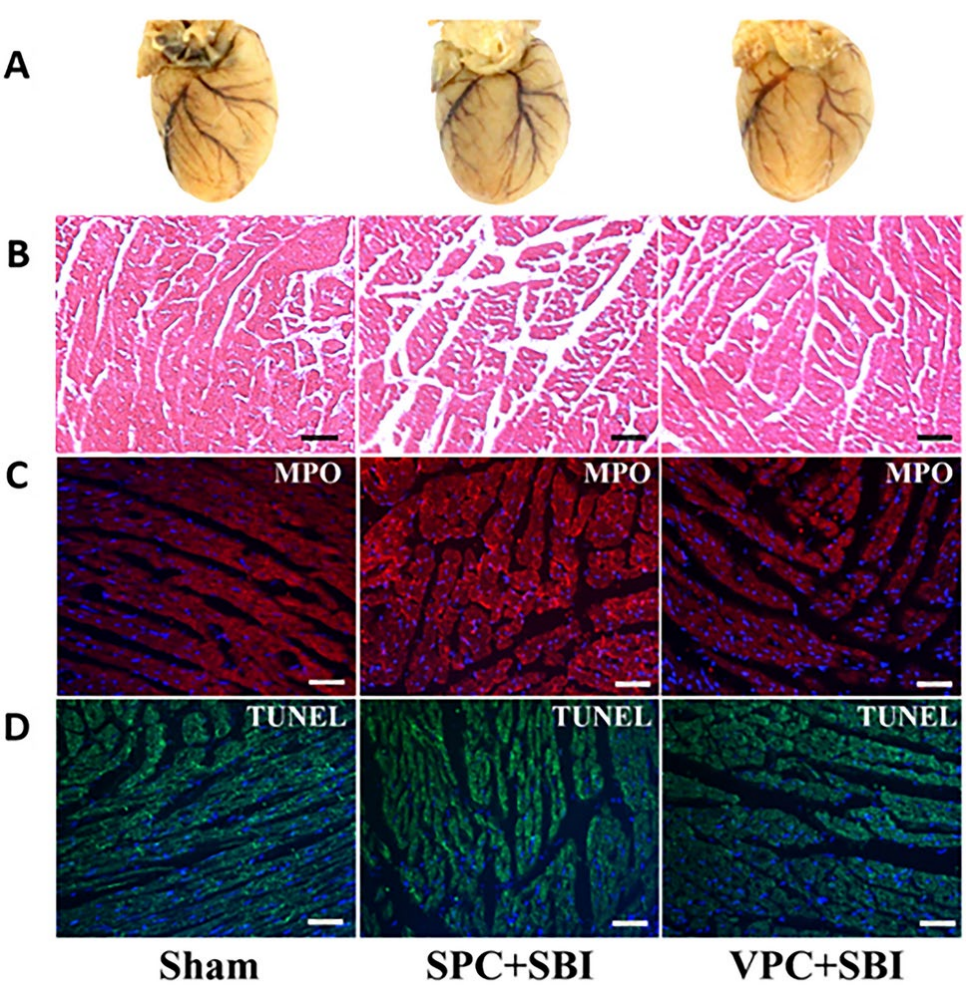
Effect of venom preconditioning (VPC) on the cardiac muscles 24 h after SBI. **(A)** Gross cardiac shape, **(B)** H&E staining, scale bar = 10 μm, **(C)** MPO staining, scale bar = 50 μm and **(D)** TUNEL staining, scale bar = 50 μm. There was no significant difference in cardiac morphology in the SPC + SBI and VPC + SBI groups compared to Sham. All the pictures are representative of 3 animals per group.

I﻿n Figure 7B the image for H&E staining is incorrect for the Sham group. This image was inadvertently duplicated from the image in the VPC+SBI group during final figure assembly.

The correct Figure 7 and accompanying legend appear below.

