# Supplementary material for: Naja sputatrix Venom Preconditioning Attenuates Neuroinflammation in a Rat Model of Surgical Brain Injury via PLA2/5-LOX/LTB4 Cascade Activation
Source: Sci Rep. 2017 Jul 14;7:5466. doi: 10.1038/s41598-017-05770-7 (PMC5511148; doi:10.1038/s41598-017-05770-7)
Supplement: Supplementary file 1 — Supplementary Information [file 41598_2017_5770_MOESM1_ESM.doc]

**Supplementary Information**

***Naja sputatrix* Venom Preconditioning Attenuates Neuroinflammation in a Rat Model of Surgical Brain Injury via PLA2/5-LOX/LTB4 Cascade Activation**

Yuechun Wang, PhD^1, 2^, Prativa Sherchan, PhD^1^, Lei Huang, MD^1, 3^, Onat Akyol, MD^1^, Devin W. McBride, PhD^1^, and John H. Zhang, MD, PhD^1, 3^*

^1^Department of Physiology & Pharmacology, Loma Linda University School of Medicine, Loma Linda, California, 92354, USA; ^2^Department of Physiology, Jinan University School of Medicine, Guangzhou, Guangdong Province, China; ^3^Department of Anesthesiology, Loma Linda University School of Medicine, Loma Linda, California, 92354, USA

* **Correspondence:**

John H. Zhang, MD, PhD

Department of Anesthesiology, Neurosurgery and Physiology

Loma Linda University, School of Medicine

Loma Linda, CA 92354, U.S.A.

Phone: 909 558 4723, Fax: 909 558 0119

Email: [johnzhang3910@yahoo.com](mailto:johnzhang3910@yahoo.com)

**Source of Funding:** This study was partially supported byNIH grants (NS084921, NS082184 and NS43338), Guangdong Province Science and Technology Plan Projects (2012B060300020), Central University Basic Scientific Research Professional Expenses Special Fund (101201221612427) and Guangdong medical scientific research fund (A2016010).

**Conflict of Interest:** The authors declare that no conflict of interest exists.

**Supplementary Figure S1.**

**
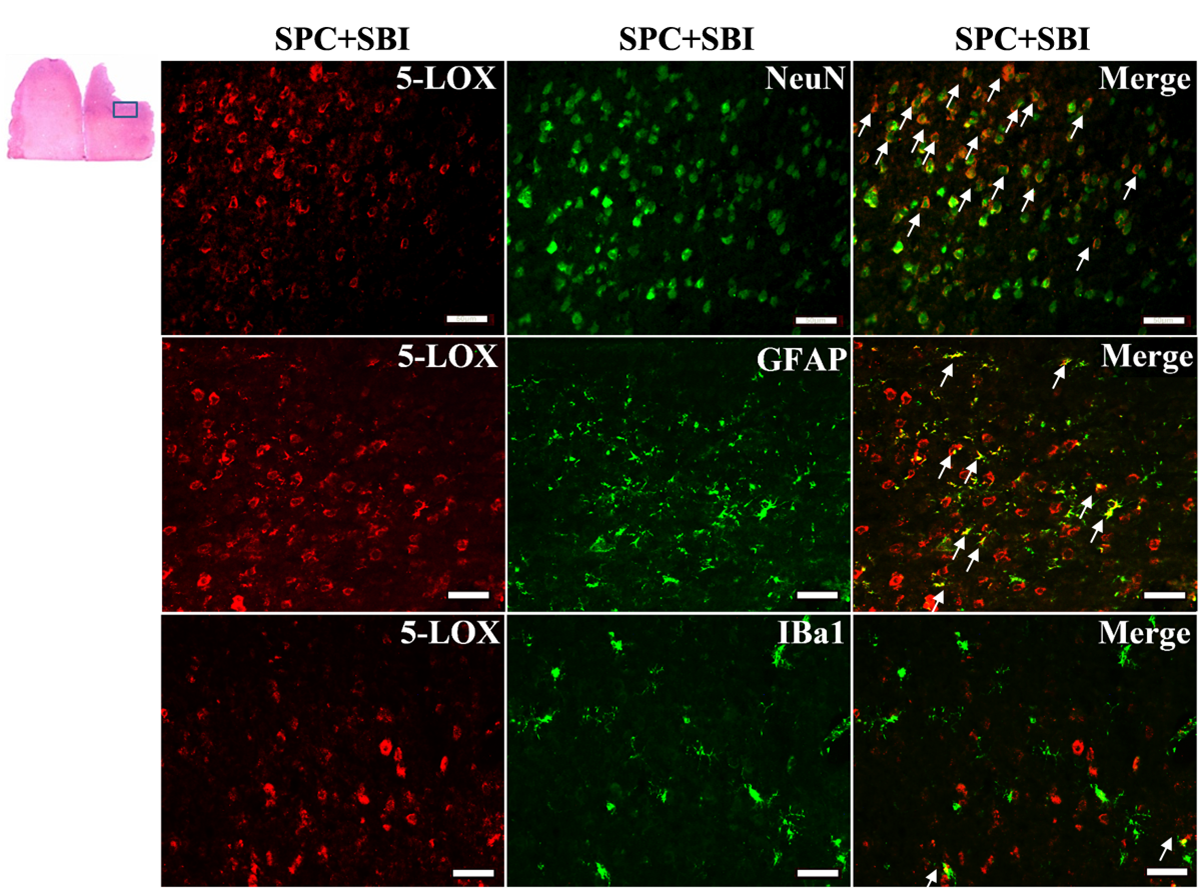
**

**Supplementary Figure S1.** Double immunofluorescence staining for cellular localization of 5-LOX at the right frontal peri-resection site in SPC+SBI group 24h after SBI. 5-LOX predominantly co-localized with the neuronal marker NeuN and to a lesser extent with the astrocyte marker GFAP but did not co-localize with the microglia marker IBa1 at the peri-resection site 24h after SBI. Arrows point to the co-localized cells. n=2/group. Scale bar=50μm.

**Supplementary Figure S2.**

**
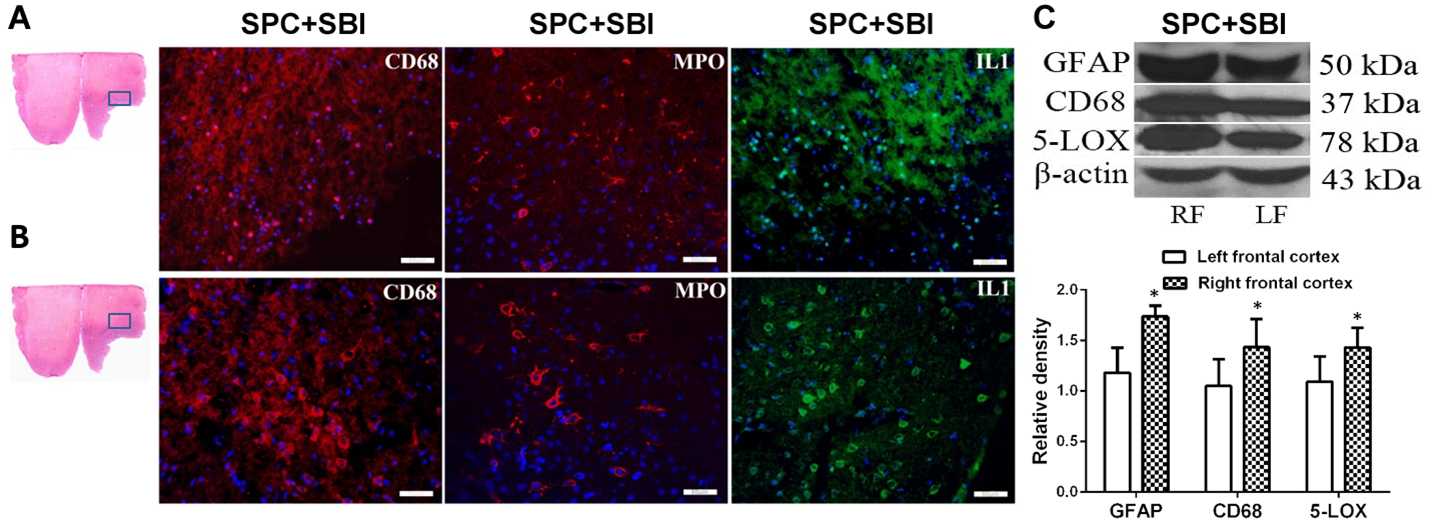
**

**Supplementary Figure S2.** Immunofluorescence staining and western blot showing inflammatory markers in the right frontal peri-resection site in SPC+SBI group 24h after SBI. Immunofluorescence staining showed inflammatory markers CD68, MPO and IL1 were distributed in the area surrounding the resection site **(A)** and extended further distant in the peri-resection area **(B)**, n=2/group. Scale bar=50μm. Brain section in the left showing within inset the region from where immunostaining pictures were taken. **(C)** Western blot showed the expression of GFAP, CD68 and 5-LOX was increased in right frontal (RF) peri-resection tissue compared to the contralateral left frontal (LF) region. *p＜0.05 vs left frontal region. Data are shown as mean ± SD. n=6/group.

**Supplementary Figure S3.**


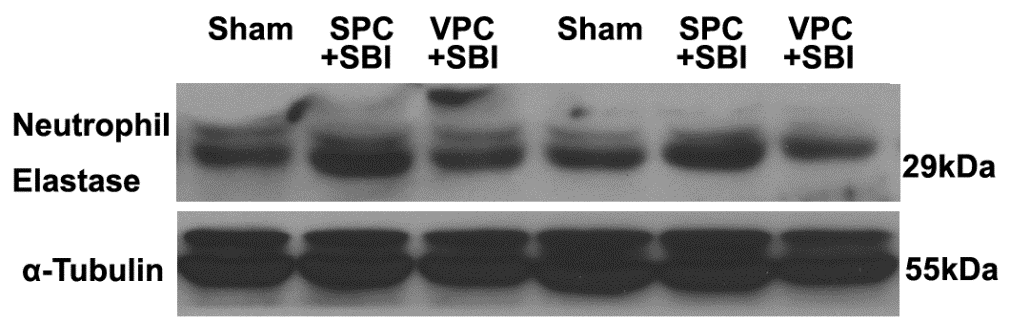


**Supplementary Figure S3.** Full length blots of the representative western blot pictures shown in Figure 4. Expression of neutrophil elastase and α-tubulin was evaluated 24h after SBI. Each lane was loaded with sample from a single animal which belonged to the group labeled in the blot. The same membrane was probed for α-tubulin as loading control.

**Supplementary Figure S4.**


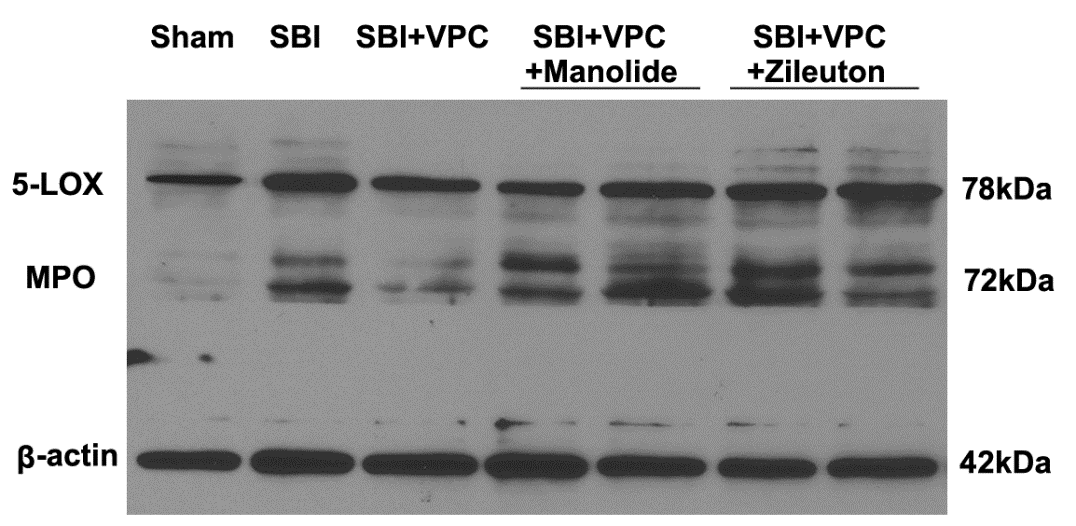


**(A)**


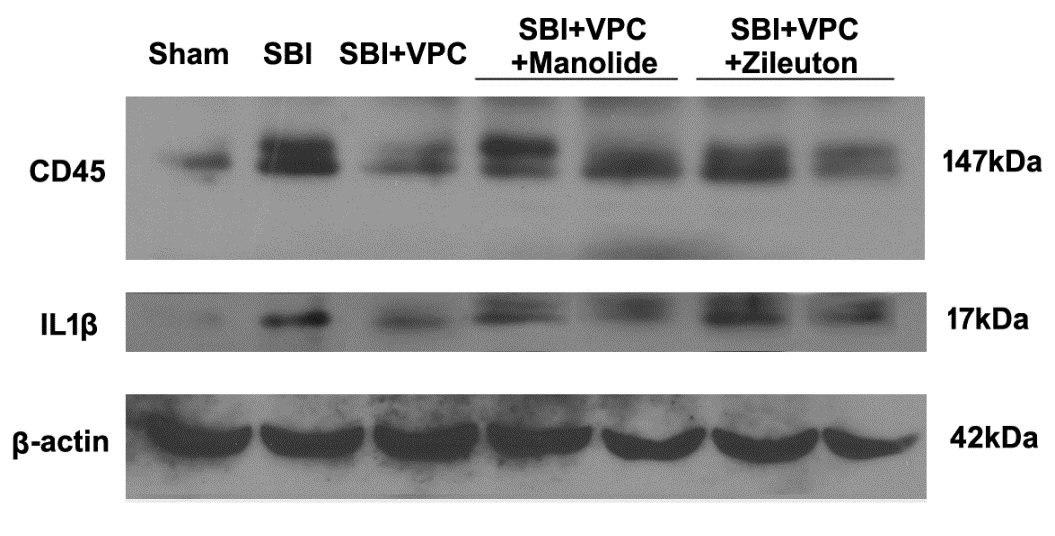


**(B)**

**Supplementary Figure S4.** Full length blots of the representative western blot pictures shown in Figure 5. Expression of 5-LOX and MPO **(A)** and CD45 and IL1-β **(B)** was evaluated 24h after SBI. Each lane was loaded with sample from a single animal which belong to the group labeled in the blot. The same membrane was probed for β-actin as loading control.

**Supplementary Figure S5.**


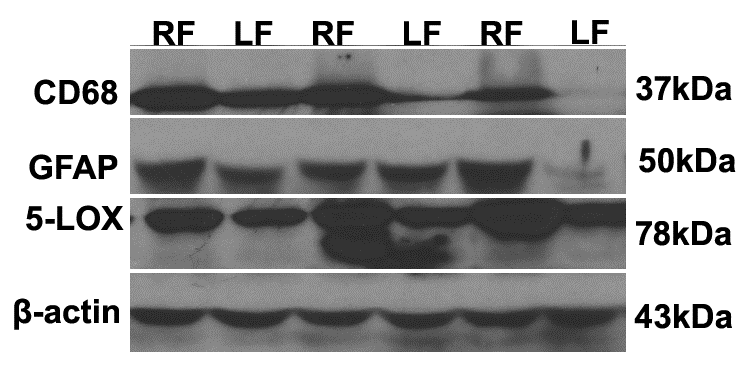


**Supplementary Figure S5.** Full length blots for representative western blot pictures shown in Supplementary Figure S2. Each lane was loaded with sample from a single animal which belonged SPC+SBI group. The same membrane was probed for β-actin as loading control. The expressions of CD68, GFAP, 5-LOX and β-actin were evaluated 24h after SBI. Abbreviations: RF (right frontal lobe), LF (left frontal lobe).
